# Retinal changes in patients with idiopathic inflammatory myopathies: A case-control study in the MyoCite cohort

**DOI:** 10.3389/fmed.2022.1065960

**Published:** 2022-11-30

**Authors:** Rachna Aggarwal, R. Naveen, Darpan Thakare, Rohit Shahi, Anamika Kumari Anuja, Ahmad Husain, Maryam Abbasi, Upendra Rathore, Vikas Agarwal, Latika Gupta

**Affiliations:** ^1^Department of Ophthalmology, Sanjay Gandhi Postgraduate Institute of Medical Sciences Lucknow, Lucknow, India; ^2^Department of Clinical Immunology and Rheumatology, Sanjay Gandhi Postgraduate Institute of Medical Sciences Lucknow, Lucknow, India; ^3^Dubai Medical College, Dubai, United Arab Emirates; ^4^Department of Rheumatology, Royal Wolverhampton Hospitals NHS Trust, Wolverhampton, United Kingdom; ^5^City Hospital, Sandwell and West Birmingham Hospitals NHS Trust, Birmingham, United Kingdom; ^6^Division of Musculoskeletal and Dermatological Sciences, Centre for Musculoskeletal Research, School of Biological Sciences, The University of Manchester, Manchester, United Kingdom

**Keywords:** retina, idiopathic inflammatory myositis, myopathy, vasculopathy, vascular health

## Abstract

**Background:**

Retinal changes are the window to systemic vasculature. Therefore, we explored retinal changes in patients with Idiopathic inflammatory myopathies (IIM) as a surrogate for vascular health.

**Methods:**

Adult and Juvenile IIM patients (2017 ACR/EULAR criteria), visiting a tertiary care center in 2021 were enrolled for detailed ophthalmic examination in comparison with healthy controls (HC). Patients with conditions that precluded thorough posterior chamber examination were excluded. Scale variables are expressed as median (IQR). Multivariate analysis (binary logistic regression-BLR) was conducted, adjusting for age, gender, and comorbidities besides factors significant in univariate analysis.

**Results:**

43 patients with IIM [31 females; age 36 (23–45) years; disease duration 5.5 (2-12) months] were enrolled for participation. DM (44%) was the most common diagnosis. IIM patients exhibited frequent attenuation of retinal vessels (32.6 vs. 4.3%, *p* < 0.001), AV nicking (14 vs. 2.2%, *p* = 0.053), and vascular tortuosity (18.6 vs. 2.2%*, p* = 0.012), besides decreased visual acuity (53.5 vs. 10.9%, p<0.001) and immature cataracts (34.9 vs. 2.2%, *p* < 0.001). Attenuation of vessels [OR 10.9 (1.7–71)*, p* = 0.004] emerged as significantly different from HC after adjusting for covariates in BLR. Notably, adults with IIM were more predisposed to retinal abnormalities [21 (57%) vs. 1 (16%)*, p* = 0.068], especially attenuation of vessels [14(38%) vs. 0(0)*, p* = 0.067] than jIIM. However, no difference was found in retinal features amongst the subtypes of adult IIM, nor did they correlate with MDAAT, MDI, or HAQ-DI.

**Conclusion:**

Retinal microvasculopathy and diminution of vision occur in nearly one-thirds to half of the patients with IIM. Microvasculopathy occurs across subtypes of IIM, and more so in adults, calling for further investigation as a surrogate for damage assessment and potentially even systemic vascular health.

## Introduction

The idiopathic inflammatory myopathies (IIMs) are a rare group of disorders with the shared manifestation of proximal muscle weakness and cell infiltrates in skeletal muscles. These diseases include polymyositis (PM), dermatomyositis (DM), inclusion body myositis (IBM), amyopathic dermatomyositis (ADM) in adults, and juvenile dermatomyositis (JDM) in children (1).

Ocular involvement in IIM includes a wide variety of manifestations involving both anterior and posterior segment pathology, however, literature is limited and evidence of ocular manifestations in IIM stems mostly from finite case studies and reports (2). Heliotrope rash, a characteristic violaceous discoloration of eyelids in DM has been the most widely demonstrable finding. Current reports of anterior segment involvement include conjunctivitis, chemosis, iritis, glaucoma and episcleritis, ptosis of eyelids, diplopia, strabismus, and nystagmus (3). Ocular muscles in DM remain unaffected even in advanced or untreated cases. Eyelid and lens abnormalities are frequently seen in patients with JDM, whereas retinopathy is rare in such patients (4).

Retinal involvement in PM and DM was first described by Bruce in 1938, followed by several case reports later on describing Purtscher-like retinopathies, unilateral retinal pigment epithelial (RPE) detachment, serous retinal detachment, cotton wool spots and optic atrophy (3, 5, 6). Retinopathy usually resolves completely without complications but can also lead to lasting and intense visual loss, with the development of retinal neovascularization. Other rare manifestations include Elschnig's spots indicating choriocapillary infarction, diffuse pallor of the optic disc, macular hemorrhage, or macular edema (6, 7). It has been postulated that systemic vasculitis which is common in JDM may put children at an increased risk of associated retinopathy (8). Keeping these sparse reports in mind, and the fact that the retinal vasculature can be a window into the health of microvasculature in the body in general, we thought it imperative to explore this facet in IIM. It is also noteworthy that diabetes and hypertension are common comorbidities in IIM, and may also contribute to retinal involvement and damage independent of IIM.

For this case-control study, patients with IIM from the MyoCite cohort were compared with healthy controls. Due to the heterogenous nature of IIM, we also explored the differences in ocular manifestations between various subtypes of IIM in adults, as well as juvenile dermatomyositis (JDM) in children. Furthermore, we aimed to identify if retinal involvement is associated with a worse prognosis or indicative of the severity of the disease, as early identification of microvasculature abnormalities in the eye may allow for timely management of vasculopathy, as well as introducing new avenues of exploration of disease activity.

## Methodology

The MyoCite dataset is a prospectively enrolled institutional review board approved study with an ongoing (2017–2021) recruitment of patients meeting the IIM ACR/EULAR classification criteria, or diagnosis of IIM as per two rheumatologists as previously described (9, 10). Clinical data is collected on a predesigned and validated detailed case record form, with clear definitions of individual organ involvement, and clinical features as previously detailed. Patients in the cohort are regularly followed up at 3–6-month intervals and core set measures including MMT8 (manual muscle testing), MDAAT (myositis disease activity assessment test), MDI (myositis damage index), and HAQ-DI (health assessment score damage index) are recorded (10–13).

### Antibody testing

Myositis specific and associated autoantibodies (MSA and MAA), and anti-nuclear autoantibodies are tested in a centralized laboratory. MSAs/MAAs are tested by line immunoassay (G4, Euro-Immune, Lubeck, Germany). Those with 2+ or more are considered positive. Anti-Nuclear Antibody (ANA) is tested by Immunofluorescence assay (IFA) using Hep-2010 cell line at a dilution of 1:100.

### Ophthalmic examination

All consenting adult and pediatric patients diagnosed with IIM that visited the center in the first half of 2021 underwent a comprehensive ophthalmic examination by two ophthalmologists which included the assessment of visual acuity, intraocular pressure with applanation tonometer, torchlight, slit lamp, and fundus examination. Any discordance was resolved by mutual consensus. A detailed case record form (CRF) was devised to obtain patient details (Supplementary Table 1). The form underwent eight rounds of revision (by RA, LG, RS) and was pilot tested in 10 individuals before being finalized. Exclusion criteria were listed at the CRF entry point.

The detailed ocular parameters on which participants were surveyed included ocular symptoms (redness, sicca, pain, headache), 16 ocular signs such as swelling of the eyelids, ptosis, proptosis, diplopia, orbital tenderness, pupil RAPD, episcleritis and scleritis, conjunctival signs (conjunctival injection, chemosis), uveitis, vitritis, immature cataracts, exacerbation of pain on eye movement, restriction of extraocular muscle movement and presence of any extraocular movements.

Additionally, patients were surveyed on specific retinal symptoms such as diminution of vision, flashes of light, floaters, scotoma, and metamorphopsia. A detailed retinal examination was conducted with the principal intention of detecting capillary-level vascular abnormalities including infarction, capillary closure, oedema, or occlusion. Specific signs included decreased visual acuity; disc, intraretinal, subretinal or macular hemorrhage; perivascular sheathing, microaneurysms, attenuation of vessels, AV nicking, vascular tortuosity, intraretinal infiltrate, copper wire or silver wire appearance, cotton wool spots, presence of hard exudates, as well as ischemia, necrosis or peri phlebitis of the retina, and disc pathologies such as drusen, oedema, or atrophy, amongst other signs. Age and gender matched healthy controls from the general population were similarly evaluated for the presence of visual abnormalities.

### Exclusion criteria

Patients with conditions that may preclude thorough posterior chamber examination, such as mature cataracts, corneal opacities, history of eye trauma within 4 weeks of examination, as well as non-consenting patients were excluded from the analysis.

### Statistical analysis

Demographic, clinical, and laboratory data of IIM, HC, and IIM subtypes were compared. Due to limited numbers, subtypes analysis was limited to DM vs. other IIM subtypes. The prevalence of various ocular and retinal signs and symptoms was assessed. Retinal changes were specifically compared between the groups. Adults and children were described separately.

Non-parametric tests were applied. Only descriptive statistics were used for groups with a sample size of < 5. All data are expressed as median and IQR. *P* < 0.05 is deemed as statistically significant and all reported values were 2-sided. SPSS version 20 was used for analysis. Assessment of data distribution and univariate analysis was conducted using Student's *t*-test or Mann Whitney U tests for continuous variables, and Chi square test and Fisher's exact for categorical variables. Comparisons are made as binary groups and multivariate analysis was done using binary logistic regression with the inclusion of statistically significant and clinically important variables. Pearson r correlation between retinal abnormality and MDAAT, MDI, and HAQ-DI was conducted.

## Results

### Patients characteristics

A total of 43 patients with IIM and 46 healthy controls were included in the study. IIM patients (31 females, 10 males) of median age 36 (23–45) years, and disease duration 5.5 (2–12) months were enrolled. Also, DM (*n* = 19, 44%) was the most common subtypes of IIM, followed by OM (*n* = 8, 18%), ASSD (*n* = 6, 14%), and PM (*n* = 4, 9%). Six JDM patients were also enrolled in the study (14%). Three IIM patients were excluded as they had either mature cataract or corneal opacities which precluded further posterior segment evaluation.

Muscle weakness and cutaneous rash were the predominant clinical features, documented in 90%, and 46% of all patients respectively (detailed patient characteristics in Table 1). MSA/MAA was positive in 70% of patients with anti-Ro 52 (14%), and anti-Mi-2 (11%) being the most common, followed by anti-MDA5 (7%) and anti-TIF1y (7%).

**Table 1:** Baseline characteristics of patients.

	**Total patients (*n =* 43)**	**Adult IIM (*n =* 37)**	**Juvenile IIM (*n =* 6)**	**Healthy controls (*n =* 46)**
Age (years)	36 (23–45)	39.5 (30–47)	8.5 (3–16)	42.5 (34–48)
Gender (Male: Female)	1:2.58 (12: 31)	(8:29)	1:0.5 (4:2)	1.7:1 (29:17)
Disease duration (months)	5.5 (2–12)	6 (2–12)	3.5 (3–14)	-
Diagnosis n (%)			6 (100)	
DM	19 (44)	19 (51)		
PM	4 (9)	4 (11)		
ASSD	6 (14)	6 (16)		
OM	8 (18)	8 (21)		
JDM	6 (14)	-		
Clinical characteristics n (%)				-
ILD	13 (30)	11 (29)	2 (33)	
Arthritis	12 (28)	10 (27)	2 (33)	
Muscle weakness	39 (90)	33 (89)	6 (100)	
Dysphagia	7 (16)	6 (16)	1 (17)	
Dysphonia	1 (2)	1 (3)	0 (0)	
Skin rash (any of the classic DM rashes)^*^	20 (46)	15 (40)	5 (83)	
Raynaud's	9 (21)	9 (24)	0 (0)	
Calcinosis	3 (7)	2 (5)	1 (17)	
Skin ulcers	7 (16)	3 (8)	4 (67)	
Periungual erythema	4 (9)	3 (8)	1 (17)	
Cuticular overgrowth	12 (28)	8 (21)	4 (67)	
MSA/MAA n (%)				-
Negative	13 (30)	10 (27)	3 (50)	
Anti-Mi2	5 (11)	5 (13)	-	
Anti-TIF1y	3 (7)	1 (3)	2 (33)	
Anti-MDA5	3 (7)	2 (5)	1 (17)	
Anti-NXP2	2 (4)	2 (5)	-	
Anti-SAE1	1 (2)	1 (3)	-	
Anti-Jo1	2 (4)	2 (5)	-	
Other ARS	1 (2)	1 (3)	-	
Anti-Ro 52	6 (14)	6 (16)	-	
Anti-Ku	2 (4)	2 (5)	-	
MYOACT (0-60)	0 (0–0)	0 (0–0)	0 (0–0)	-
MDI Severity of damage score (0-110)	0 (0–3)	0 (0–3)	0 (0–0)	-
Comorbidities				
Diabetes	3	3	0	4
Hypertension	3	3	0	2
CKD	0	0	0	0
CLD	0	0	0	0
CAD	0	0	0	0
Malignancy	1	1	0	0
Smoker/tobacco chewer	4	4	0	0
Alcohol intake	3	3	0	0

ASSD, Anti-synthetase syndrome; ARS, Aminoacyl tRNA synthetase antibody; DM, Dermatomyositis; PM, Polymyositis; JDM, Juvenile dermatomyositis; OM, Overlap myositis; ILD, Interstitial lung disease; MYOACT, Myositis Disease Activity Assessment Visual Analog Scale; MDI, severity of damage score is sum of 11 VAS scores to assess damage.

^*^Gottron's sign and papule, Heliotrope, V sign, Shawl sign, facial rash, Linear erythema, Holster sign.

Among 43 patients enrolled in the study, a higher proportion (23.3%) reported visual disturbances than controls (10.9%), although the numbers were not statistically higher. However, upon formal vision testing, a higher proportion of IIM (53.5%) had decreased visual acuity than controls (10.9%, *p* < 0.001).

### Retinal vascular changes are more common in IIM than in healthy controls

Attenuation of retinal vessels was more often seen in IIM patients (32.6%) as compared with healthy controls (4.3%, *p* < 0.001) (multivariate OR: 10; 95%CI: 1.7–71, *p* = 0.004). Other retinal vascular abnormalities such as arteriovenous nicking (14 vs. 2.2%, *p* = 0.053), and vascular tortuosity (18.6 vs. 2.2%, *p* = 0.012) were also more prevalent in IIM patients compared with healthy controls.

Retinal changes such as disc atrophy, retinal periphlebitis, and cotton wool spots were reported in 2.3% of IIM and were absent in the HC. Perivascular sheathing was found in 4.6% of IIM and 2.2% of HC (Table 2). Severe retinal changes including disc oedema, disc hemorrhage, macular hemorrhage, intra retinal infiltrates, sub-retinal hemorrhage, silver wire appearance, retinal necrosis, and retinal ischemia were absent in both myositis and the healthy control groups.

**Table 2:** Distribution of ocular symptoms and retinal signs and symptoms between IIM patients and healthy controls.

**Variables**	**IIM (*N =* 43)**	**Healthy (*N =* 46)**	**p**
**Symptoms related to retina**			
Diminution of vision	10 (23.3)	5 (10.9)	0.119
Flashes of Light	0	0	–
Floaters	0	0	–
Scotoma	0	0	–
Metamorphopsia	0	0	–
**Retinal signs**			
Decreased visual acuity	23 (53.5)	5 (10.9)	< 0.001
Disc drusens	1 (2.3)	1 (2.2)	0.999
Disc oedema	0	0	–
Disc hemorrhage	0	0	–
Disc atrophy	1 (2.3)	0	0.483
Macular hemorrhage	0	0	–
Perivascular sheathing	2 (4.6)	1 (2.2)	0.608
Retinal periphlebitis	1 (2.3)	0	0.483
Cotton wool spot	1 (2.3)	0	0.483
Hard exudate	0	1 (2.2)	0.999
Intra retinal infiltrate	0	0	–
Sub retinal hemorrhage	0	0	–
Intra retinal hemorrhage	1 (2.3)	1 (2.2)	0.999
Microaneurysm	0	1 (2.2)	–
Attenuation of vessel	14 (32.6)	2 (4.3)	< 0.001
AV nicking	6 (14)	1 (2.2)	0.053
Vascular tortuosity	8 (18.6)	1 (2.2)	0.012
Copper wire appearance	0	1 (2.2)	0.483
Silver wire appearance	0	0	–
Retinal necrosis	0	0	–
Retinal Ischaemia	0	0	–
Other	4 (9.3)	0	0.051

AV, Arteriovenous.

Chi square test / Fisher exact test were used.

P < 0.05 significant.

### Retinal changes amongst different IIM

No difference was observed in any retinal abnormality between DM and other IIM subtypes, although numbers were limited to draw firm conclusions. Adult IIM (*n* = 37) exhibited trends to higher retinal abnormalities [21 (57%) vs. 1 (16%), p 0.068] and attenuation of vessels [14 (38%) vs. 0 (0), *p* 0.067] than JDM.

No differences were found in patients with and without comorbidities as far as retinal abnormalities were concerned. Weight loss was frequently reported in those with retinal abnormalities [62 vs. 13%, OR (95%CI) 10 (1.8–59), *p* 0.004].

### Other ocular signs and symptoms in IIM patients and healthy controls

Immature cataracts were significantly more common in IIM than HC (34.9 vs. 2.2%, *p* < 0.001) (Supplementary Table 1). Sicca, swelling of eyelids, episcleritis, redness of eyes, and vitritis were similar to HC and were rarely seen.

Pain, flashes of light, floaters, scotoma, metamorphopsia, ptosis, proptosis, conjunctival injection, chemosis, scleritis, abnormal pupil reaction, diplopia, orbital tenderness, exacerbation of pain on eye movement and restriction of extraocular muscle movement were not reported in either group.

### Correlation of retinal abnormality with disease activity and damage

The presence of any retinal abnormalities did not correlate with disease activity measure (MDAAT r −0.182, *p* 0.243) or damage scores (MDI r 0.199, *p* 0.201) or HAQ-DI (r −0.298, p 0.116). Neither did it correlate with muscle enzymes (CPK r −0.007, p 0.166; LDH r −0.131, p 0.571).

## Discussion

Our key observations included poor vision in nearly half of the patients studied, and microvascular changes in nearly one-third, the changes being more common in adults with IIM. The retinal vascular changes included attenuation of retinal vessels, arteriovenous nicking, and vascular tortuosity. Immature cataracts and decreased vision were also more prevalent in IIM than HC, despite the exclusion of patients with marked visual impairment. Further the retinal changes in IIM were consistent irrespective of presence of comorbidities like diabetes or hypertension.

Notably, we did not find any difference between the various subtypes of IIM, possibly due to small sample size. Comorbidities especially diabetes and hypertension did not seem to impact the prevalence of retinal changes, suggesting that these changes are inherent to the disease and potentially surrogate for systemic vascular health. We couldn't find an association between these changes with activity or damage assessment. Nevertheless, the retinal microvascular changes were more frequent in patients with a history of weight loss, creating the case for a potential association with systemic inflammation.

This is the first structured case-control study to explore retinal changes in IIM. Previous studies affirm the presence of exhibit retinal vascular changes such as cotton wool spots, retinal hemorrhages, and vascular tortuosity in up to 10% of SLE patients and a third of systemic sclerosis patients respectively (14, 15). The comparable findings of retinal abnormalities in IIM patients may be attributed to either accelerated atherosclerosis, vasculitis, or vasculopathy. Whilst vasculitis is more often associated with jDM and anti-MDA5 subtypes of adult IIM, accelerated atherosclerosis seems a more likely possibility considering emerging evidence on poor cardiovascular health in patients with IIM (16, 17). The presence of cardiovascular comorbidities can entail additional risk for the latter, though this association could not be confirmed in the current study.

Early retinal involvement in IIM has been plausibly linked to retinal endothelial inflammation or dysfunction affecting the retinal microvasculature in published literature from the 1980s (8). This theory can be supported by the significant attenuation of arterioles and arteriovenous nicking noted in IIM patients compared with controls in our study. Both of these findings are early signs of hypertensive retinopathy; however, no significant difference was found in subjects with and without comorbidities. We hope that large prospective multicenter studies with structured damage assessment can address this possibility (18).

Increased vessel tortuosity was also evident in patients with IIM. Vascular tortuosity has been attributed to the altered tone of smooth vascular muscle in response to metabolites such as lactic acid, blood gases, and prostaglandins (19). Tortuosity is becoming an increasingly interesting avenue as an indicator of the relevant vascular and non-vascular disease, with high prognostic performance with both assessments by ophthalmologists and by computational retinal image analysis. This may provide non-invasive prognostic and diagnostic use in IIM (20).

The comparison of adult and juvenile IIM patients revealed a higher prevalence of retinal abnormality and attenuation of vessels in adult IIM, though not statistically significant, possibly due to limited numbers. Emerging evidence suggests accelerated atherosclerosis in these children from calcinosis-mediated NETosis and dyslipidaemia (21, 22). Neutrophil activation in JDM accelerates endothelial damage and promotes oxidative modification of HDLs to a pro-thrombotic form. JDM patients also exhibit low levels of HDL and elevated calprotectin, thus predisposing JDM patients to atherosclerosis and cardiovascular disease (21). The fine correlation of inflammation and vasculopathy in JDM may present opportunities to detect vascular pathology in the form of retinal changes early in the disease and reduce cardiovascular risk in patients by targeting therapy at calcinosis and NETosis specifically.

We did not find an association between retinal changes and activity or damage assessment in IIM. Exclusion of patients with severe cataracts may potentially have excluded those with higher damage scores or HAQ-DI, introducing an element of bias into the analysis (7). Assessment of nail fold capillaroscopy and triangulation with retinal vascular health is an approach explored by researchers previously in systemic sclerosis (21).

Our study is limited in the number of subjects enrolled and heterogeneous sample. Further, the ophthalmologists were not blinded to patients versus controls. We hope that larger cohort studies can triangulate fundus fluorescein angiography pictures with retinal assessment to substantiate this aspect of vascular health in IIM. These findings may carry important implications for damage assessment in myositis, as retinal involvement may be a window into the health of systemic vasculature. Our preliminary observations, although limited in sample size and heterogeneity of the cohort, build a case to explore this in larger multicentric studies.

In conclusion, our study suggests that retinal microvasculopathy and diminution of vision are fairly common in IIM patients and could substantiate early damage and cardiovascular health assessment. Microvasculopathy occurs independent of comorbidities and subtypes of IIM, creating a case for further exploration.

## Data availability statement

All data pertaining to this study is available in the main file and as Supplementary material.

## Author contributions

All authors were involved in ideation, data collection, and manuscript preparation, agree with the submitted version of the manuscript, take responsibility for the content of the entire manuscript, and affirm that any queries related to any aspect of the same are appropriately managed.

## Funding

The MyoCite biobank was supported by the APLAR research grant.

## Conflict of interest

The authors declare that the research was conducted in the absence of any commercial or financial relationships that could be construed as a potential conflict of interest.

## Publisher's note

All claims expressed in this article are solely those of the authors and do not necessarily represent those of their affiliated organizations, or those of the publisher, the editors and the reviewers. Any product that may be evaluated in this article, or claim that may be made by its manufacturer, is not guaranteed or endorsed by the publisher.
