# Supplementary material for: Retinal changes in patients with idiopathic inflammatory myopathies: A case-control study in the MyoCite cohort
Source: Front Med (Lausanne). 2022 Nov 30;9:1065960. doi: 10.3389/fmed.2022.1065960 (PMC9747771; doi:10.3389/fmed.2022.1065960)
Supplement: Supplementary file 1 [file Table_1.docx]

| Supplementary Table 1. Distribution of other Ocular symptoms among IIM patients and healthy controls | | | |
| --- | --- | --- | --- |
| Variables | Patients (N=43) | Healthy Controls (N=46) | p |
| **Ocular Symptoms** |  |  |  |
| Redness | 0 | 1(2.2) | -- |
| Sicca | 1 (2.3) | 0 | -- |
| Pain | 0 | 0 | -- |
| Headache | 1 (2.3) | 0 | -- |
| **Ocular Signs** |  |  |  |
| Swelling of eyelid | 1 (2.3) | 0 | -- |
| Ptosis | 0 | 0 | -- |
| Proptosis | 0 | 0 | -- |
| Conjunctival injection | 0 | 0 | -- |
| Chemosis | 0 | 0 | -- |
| Episcleritis | 1 (2.3) | 0 | -- |
| Scleritis | 0 | 0 | -- |
| Pupil RAPD | 0 | 0 | -- |
| Uveitis | 1(2.3) | 1(2.2) | -- |
| Diplopia | 0 | 0 | -- |
| Orbital tenderness | 0 | 0 | -- |
| Exacerbation of pain on eye movement | 0 | 0 | -- |
| Restriction of extra-ocular muscle movement | 0 | 0 | -- |
| Vitritis | 1 (2.3) | 0 | -- |
| Extra Ocular moments | 0 | 0 | -- |
| Immature cataract | 15(34.9) | 1(2.2) | <0.001 |
| RAPD Relative afferent pupillary defect, Chi square test / Fisher exact test were used. **P<0.05 significant.** Those with small numbers, OR, p value were not calculated. | | | |
